# Post-Esophagectomy Tube Feeding: A Retrospective Comparison of Jejunostomy and a Novel Gastrostomy Feeding Approach

**DOI:** 10.1371/journal.pone.0089190

**Published:** 2014-03-21

**Authors:** Kenan Huang, Bin Wu, Xinyu Ding, Zhifei Xu, Hua Tang

**Affiliations:** Department of Thoracic and Cardiovascular Surgery, Shanghai Changzheng Hospital, The Second Military Medical University, Shanghai, China; Roswell Park Cancer Institute, United States of America

## Abstract

**Background:**

McKeown-type esophagectomy combined with retrosternal reconstruction is a common surgical treatment for esophageal cancer. Various enteral feeding options are available post-esophagectomy, but no definitive preference exists.

**Method:**

“Retrosternal Route Gastrostomy Feeding (RGF)” was developed as an alternative enteral feeding approach that requires few additional surgical interventions. RGF is based on McKeown-type esophagectomy. We retrospectively compared RGF (n = 121) to jejunostomy feeding (JF) (n = 153) in 274 patients at the Department of Cardiothoracic Surgery in Changzheng Hospital (Shanghai, China) between June 2008 and Sept. 2012. Data pertaining to efficacy and procedural complications were compared among patients.

**Results:**

RGF had a significantly shorter postoperative hospital stay (11 vs. 15 days, p<0.001) and time to removal of the feeding tube (9 vs. 14 days, p<0.001) compared to JF. Bowel obstruction (0.0% vs. 7.2% p = 0.003), abdominal distension (9.1% vs. 19% p = 0.022), and the occurrence of pneumonia (11.6% vs. 26.1% p = 0.003) were significantly lower in the RGF group. Feeding tube related complications and the associated morbidity rate were reduced in the RGF group. The two groups had similar tolerance to surgery.

**Conclusion:**

Our data suggests that RGF is a safe post-esophagectomy enteral feeding alternative to JF.

## Introduction

Esophagectomy is the preferred surgical approach for esophageal cancer treatment. After surgery, patients require nutrition to support recovery and their stressed immune system [1], [2]. Several feeding methods have been used for esophageal cancer patients in the postoperative period. Jejunostomy tube feeding is commonly used to support the nutritional and medicinal requirements in these patients [3]. However, jejunostomy is associated with serious complications, such as volvulus, internal hernia, bowel obstruction, and even mortality [3], [4]. Tri-lumen tube feeding has been widely used in patients after esophageal cancer surgery, but this method is limited by obstruction of the thin feeding tube, and throat pain caused by long-term tube insertion. Is there a better way for postoperative nutrition?

The concept of 3-field esophagectomy was introduced by McKeown [4], who first described transthoracic esophagectomy with cervical anastomosis. For patients with regional esophageal cancer, subtotal esophagectomy with a thoracic-abdominal-cervical incision (McKeown-type esophagectomy) combined with extensive lymphadenectomy is generally recognized as an optimal treatment in terms of long-term survival [5]–[9]. In esophageal carcinoma patients, retrosternal reconstruction is usually performed as the procedure is reported to have several advantages, including prevention of tumor recurrence and avoidance of conduit irradiation. If postoperative radiation therapy is needed for recurrent disease, efficient drainage for anastomotic leaks and ease of reoperation for anastomotic strictures are essential [10].

In the current study, we developed an enteral feeding approach combining McKeown-type esophagectomy with retrosternal reconstruction. We compared the approach with traditional jejunostomy in terms of efficacy and complications.

## Patients and Methods

### 1. General data

This retrospective study was approved by the Institutional Review Board of Changzheng Hospital (Shanghai, China), and all participants provided written informed consent. A total of 274 patients underwent three-incision esophagectomy (right chest/belly/left neck) at the Hospital between June 2008 and September 2012, after which digestive tract reconstruction was achieved by a retrosternally positioned gastric tube. The trial included 232 males and 42 females aged 44–78 years. 66 patients had carcinoma in the upper part of the esophagus; 49 patients had carcinoma in the distal part of the esophagus; and 159 patients had carcinoma in the middle part of esophagus. The patients were divided into two groups in a randomized manner: the novel Retrosternal Route Gastrostomy Feeding group (RGF group, n = 121) and the Jejunostomy Feeding group (JF group n = 153). No patient received radiotherapy or chemotherapy before surgery. Patients' demographic data are shown in Table 1. There were no significant differences in the clinical backgrounds of the patients among the two groups.

**Table 1:** Patients' demographic data (n, %).

	RGF(n = 121)	JF(n = 153)	p Value
Male	103(85.1%)	129(84.3%)	0.853
Age (mean±SD, y)	61.5±6.5	61.8±7.2	0.700
History of tobacco use	94(77.7%)	121(79.1%)	0.780
Underlying disease			
Diabetes mellitus	38(31.4%)	52(34.0%)	0.651
Hypertension	46(38.0%)	60(39.2%)	0.840
Coronary artery disease	20(16.5%)	27(17.6%)	0.807
Neurologic dysfunction	14(11.6%)	19(12.4%)	0.830
TNM stage			
I	20(16.5%)	38(24.8%)	0.095
II	40(33.1%)	60(39.2%)	0.293
III	45(37.2%)	43(28.1%)	0.110
IV	16(13.2%)	22(14.4%)	0.783
Postoperative hospitalization (M, range; d)	11(8–31)	15(9–40)	<0.001
Days of keeping feeding tube (M, range; d)	9(7–25)	14(8–38)	<0.001
Intubation days of gastric tube	4.5±2.3	8.0±2.6	<0.001

### 2. Operative procedure

#### Retrosternal route gastrostomy

**Figure 1:**
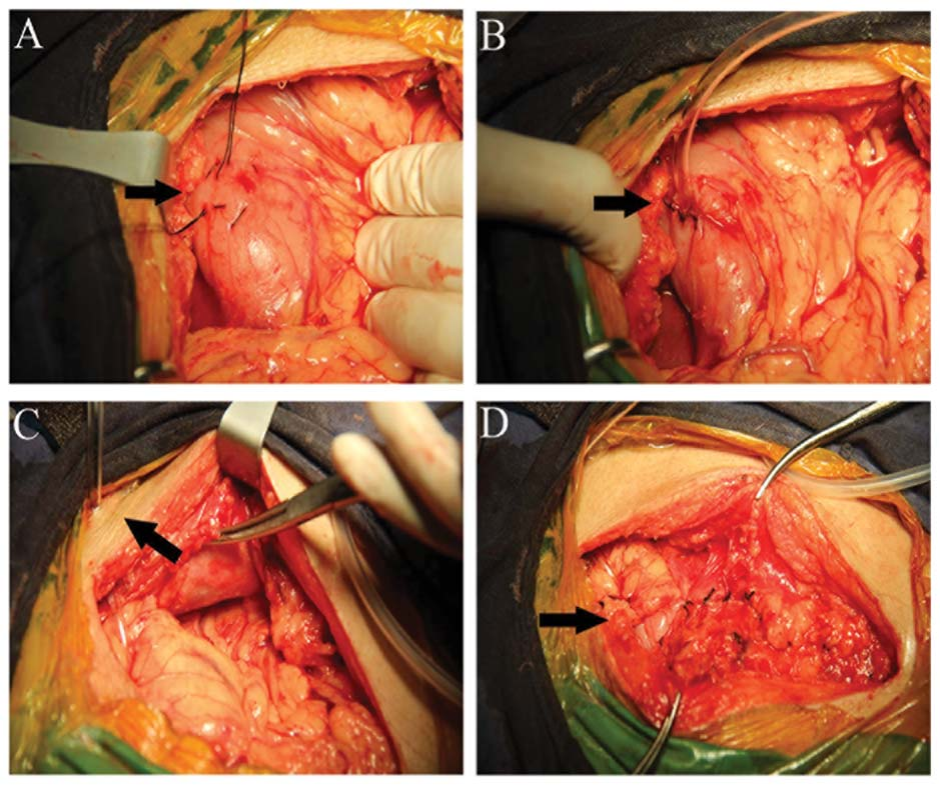
The operative procedure for retrosternal route gastrostomy. (A) The black arrow shows a purse-string suture in the anterior wall of the stomach under the xiphoid; (B) The black arrow shows the feeding tube was inserted through the incision, and 10 to 25 cm of the tube was passed aborally in the intestingal lumen; (C) The black arrow shows the feeding tubes brought out through the anterior abdominal wall; (D) The black arrow shows the omentum and the peritoneum were sutured to the gastric wall near the tube.

After the esophagus was reconstructed with a gastric tube inserted retrosternally, a 3.0–5.0-cm linear incision was made in the anterior wall of the stomach under the xiphoid. A feeding tube (16 Fr. gastric tube; length, 125 cm; diameter, 5.3 mm; TERUMO Medical Products Co., Ltd. Hangzhou, China) was inserted through the incision into the intestinal lumen to a depth of 10 to 25 cm. The feeding tube was secured in place by a double purse-string suture, embedded within the omentum, and the peritoneum was sutured to the gastric wall near the tube. Subsequently, the feeding tube was passed out through the anterior abdominal wall and secured in place (Fig. 1; Video S1)

#### Jejunostomy

Jejunostomy was performed using a standard approach. A 15 cm segment of jejunum beyond the ligament of Treitz was selected. A feeding tube (16 Fr. gastric tube; length, 125 cm; diameter, 5.3 mm) was passed through the abdominal wall, advanced 8 cm in a submucosal tunnel, and fed through the mucosa into the jejunal lumen. The catheter was advanced 20 cm and secured with an absorbable purse string suture. An additional seromuscular Witzel [11] tunnel was fashioned to overlap the catheter and the purse-string suture site. Subsequently, the jejunum was secured to the anterior abdominal wall with interrupted absorbable sutures. Nasogastric (NG) tubes were placed in all patients until the return of bowel function.

### 3. Nutrition

Enteral feeding began 6 h after esophagectomy. The regimen comprised 5% glucose solution infused at a rate of 20 ml per hour for the first 12 h, followed by infusion of Nutrison (Nutricia Export B.V, Holland) at a rate of 15 ml/h on day 1, 20 ml/h on day 2, and 25 ml/h from day 1–5. For controlled delivery, infusion pumps were used to provide the diet for 20–22 h, followed by a rest period of 2–4 h. Anastomotic leak was evaluated with the methylene blue test in all patients on post-operative day 5. For the patients without anastomotic leakage, light liquid food was initially fed. Probiotics and antimotility agents were administered in patients who developed diarrhea. Blocked catheters were treated with either normal saline flushing or relaparatomy. Ventosity was treated with adjustments to feeding speed.

### 4. Clinical index

Preoperative diagnosis; clinical stage; complications related to surgery; complications associated with the catheter, including wound infection, peritonitis, catheter displacement, and catheter blockade; digestive system complications, including bowel obstruction and abdominal distension; time to removal of the indwelling feeding tube and gastric tube; and the daily volume of gastric juice volume were recorded.

The length of the postoperative hospital stay (LOHS) was defined as the number of days from the day of operation until the date of discharge. Postoperative mortality was defined as any death during the hospital stay after surgery. Patients taking medications for hyperglycemia or hypercholesterolemia were considered diabetic and hyperlipidemic, respectively.

### 5. Statistical analysis

Statistical analysis was performed using Statistics Package for Social Science (SPSS 16.0) software. Continuous data are expressed as mean±standard deviation or median (interquartile range). Unpaired student t tests were used for comparisons between means of groups with normally distributed continuous variables. Wilcoxon rank sum tests were used for comparisons between medians of groups with skewed data. Categoric variables are expressed as percentage frequency. χ^2^ or Fisher exact tests were used to compare categoric data between groups. Multivariate logistic regression model was utilized for each dependent variable of interest to determine the predictors of postoperative pneumonia. A generalized estimating equation was used to compare daily gastric juice volume (X) between the two groups of patients: grade 1, X<100 ml; grade 2, X>100 ml–<200 ml; grade 3, X>200 ml.

## Results

Demographic data indicated that 42 (15.3%) patients were female and the mean age of patients was 61.7 (range from 44 to 78) years. The median LOHS (11 vs.15 days; p<0.001) and the median time to removal of the feeding tube (9 vs. 14 days; p<0.001) were significantly shorter in the RGF group compared to the JF group The intubation time of the gastric tube was significantly shorter in the RGF group compared to the JF group (4.5±2.3 vs. 8.0±2.6 days; p<0.001) (Table 1).

There were significantly lower incidences of digestive system complications in the RGF group compared to the JF group (bowel obstruction: 0 of 121 [0.0%] vs. 11 of 153 [7.2%], p = 0.003; abdominal distension: 11 of 121 [9.1%] vs. 29 of 153 [19%], p = 0.022). There was no significant difference in the incidence of reflux symptoms between the two groups. There were lower incidences of complications related to the feeding tube in the RGF group compared to the JF group (wound infection 2 vs. 10; peritonitis 0 vs. 11; catheter displacement 2 vs. 7; catheter blockade 4 vs. 9); the difference was significant for peritonitis (p = 0.003). There was a lower morbidity rate related to surgical complications in the RGF group compared to the JF group. There were no significant differences in the incidences of anastomotic leak, wound infection, chylothorax, and dysrhythmia; however, the difference was significant for pneumonia (14 of 121 [11.6%] vs. 40 of 153 [26.1%], p = 0.003) (Table 2).

**Table 2:** Incidence of postoperative complications between two groups (n, %).

Type of complications	RGF	JF	p Value
Surgical complications			
Wound Infection	9(7.4%)	17(11.1%)	0.303
Anastomotic Leak	5(4.1%)	10(6.5%)	0.385
Chylous Leakage	2(1.7%)	3(2.0%)	0.850
Arrbythmias	13(10.7%)	19(12.4%)	0.668
Pneumonia	14(11.6%)	40(26.1%)	0.003
Catheter related			
Wound Infection	2(1.7%)	10(6.5%)	0.050
Peritonitis	0(0.0%)	11(7.2%)	0.003
Catheter displacement	0(0.0%)	2(1.3%)	0.207
Catheter blockade	4(3.3%)	9(5.9%)	0.319
Digestive system complications			
Bowel obstruction	0(0.0%)	11(7.2%)	0.003
Abdominal distension	11(9.1%)	29(19%)	0.022
Backflow	13(10.7%)	21(13.7)	0.457

In the multivariate regression model, the intubation time of the gastric tube was the strongest predictor for postoperative pneumonia (odds ratio 8.52; 95% confidence interval 4.37–16.62). The other predictor was smoking (Table 3).

**Table 3:** Two risk factors related to postoperative pneumonia were confirmed with logistic regression analysis.

Variable	B	S.E.	Wald	df	Sig.	OR	95% CI for EXP(B)	
						Lower	Upper	
Intubation time of gastric tube	2.142	.341	39.480	1	.000	8.519	4.367	16.619
Smoking	.886	.499	3.158	1	.076	2.426	.913	6.447
Constant	−7.367	1.024	51.774	1	.000	.001		

Note: n = 274; CI = confidence interval; OR = odds ratio.

The generalized estimating equations indicated that the daily gastric juice volume of the RGF group was significantly lower than that of the JF group, except on postoperative Day 12 (Table 4).

**Table 4:** Parameter Estimation.

Parameter		B	S.E.	95% CI for EXP(B)		Hypothesis testing		
				Lower	Upper	Wald	df	Sig.
threshold	[Grade = 1.00]	2.280	.2604	1.769	2.790	76.611	1	.000
	[Grade = 2.00]	3.789	.2923	3.216	4.362	168.057	1	.000
	[Grade = 3.00]	5.182	.3145	4.565	5.798	271.473	1	.000
[Group RGF = 1.00]		−1.764	.1553	−2.068	−1.460	128.968	1	.000
[Group JF = 2.00]		0a	.	.	.	.	.	.
[Time = 1.00]		5.487	.3210	4.858	6.116	292.161	1	.000
[Time = 2.00]		5.323	.3253	4.685	5.960	267.783	1	.000
[Time = 3.00]		4.937	.3064	4.337	5.537	259.704	1	.000
[Time = 4.00]		4.534	.2963	3.954	5.115	234.249	1	.000
[Time = 5.00]		4.258	.2900	3.690	4.827	215.568	1	.000
[Time = 6.00]		3.787	.2866	3.225	4.348	174.634	1	.000
[Time = 7.00]		3.182	.2693	2.654	3.710	139.644	1	.000
[Time = 8.00]		2.321	.2614	1.809	2.833	78.833	1	.000
[Time = 9.00]		1.383	.2245	.943	1.823	37.934	1	.000
[Time = 10.00]		.486	.1544	.183	.788	9.907	1	.002
[Time = 11.00]		.160	.0899	−.016	.336	3.177	1	.075
[Time = 12.00]		0a	.	.	.	.	.	.
(scale)		1						

## Discussion

Esophagectomy is the treatment of choice for most esophageal cancer patients [12]. Providing support for patients' postoperative nutritional requirements reduces postoperative complications and has become an important part of the peri-operation period. Due to difficulty in swallowing and treatment related anorexia, esophageal cancer patients have varying degrees of dysphagia resulting in malnutrition, impaired immune function, and morbidity such as anastomotic fistula and infection [13]. The benefits of early nutritional support after esophagectomy include improved wound healing and decreased complications [14]. Enteral nutrition is also reported to help preserve gut structure and function [15], enhance gut mediated immunity [16], and is feasible in over 90% of patients undergoing gastrointestinal surgery [17]. Moreover, randomized comparisons have reported that enteral nutrition is superior to parenteral nutrition in terms of clinical outcomes [18], resulting in fewer septic complications [19], [20] and shorter lengths of hospital stay [21]. The use of enteral nutrition is often a requirement in esophagectomy patients until they can be fed by mouth

Common methods of enteral nutrition after esophago gastrectomy include indwelling NG feeding tube and jejunostomy feeding. The use of NG tubes is associated with complications [22]–[24]. The presence of a NG tube hampers effective coughing and compromises pulmonary hygiene [22]. NG tubes allow translocation of gastrointestinal flora into the upper airways causing pneumonia [23]. Disadvantages of jejunostomy include invasiveness, volvulus, internal hernia and bowel obstruction [25]–[27]. Many of these complications require re-laparotomy.

For patients with regional esophageal cancer, subtotal esophagectomy with a thoracic-abdominal-cervical incision (McKeown-type esophagectomy) combined with retrosternal reconstruction is a treatment option. Previously this approach was limited, because the surgery involved replacement of the whole stomach, which caused compression of the heart and great vessels, leading to arrhythmia. With the advent of thoracoscopy for esophageal cancer and the use of a tubular stomach, the substernal pathway has become accepted by the majority of surgeons. Furthermore, esophageal reconstruction with a tubular stomach is associated with less arrhythmia.

As part of reconstruction, a portion of the stomach is moved into the upper peritoneum. This provides a new site for enteral feeding. We investigated the efficacy of placing a feeding tube at this site. Our data show a clear advantage for RGF over JF. In 153 JF patients, we recorded a longer intubation time of the indwelling stomach tube and higher incidences of three complications, including 11 bowel obstructions, 11 cases of peritonitis, and 40 cases of pneumonia. In contrast, 121 patients that underwent RGF had no or few feeding tube or digestive tract associated complications and a lower incidence of pulmonary infection. As an added benefit, the duodenal transpyloric placement of the RGF feeding tube stimulates peristalsis, in accordance with the normal physiology and functioning of the human body.

As the RGF tube was embedded in the extraperitoneal cavity, the distance between the internal and external apertures is short (equivalent to the thickness of the abdominal wall). This reduces the risk for fistula formation. Therefore, in the incidence of serious tube related complications, the RGF tube can be removed within a week after operation while the JF tube requires two weeks.

In the early postoperative period, a gastric tube is imperative for maintaining effective decompression of the conduit. Preventing conduit distention helps avoid aspiration of gastrointestinal contents from a dilated, fluid-filled conduit [28]. NG tubes predispose to respiratory complications including aspiration, pneumonia, sinusitis, pharyngitis, and laryngitis, particularly in patients who have recently undergone a major operation [29]. RGF tubes drain by gravity and stent the pylorus, thus encouraging antegrade flow of gastric secretions. In our study, we noted a shorter time of indwelling stomach tube and a lower incidence of pulmonary infection with the use of RGF tubes.

This study has several important limitations. First, the efficacy of the novel approach in patients with varying BMIs has not proved. We have no BMI measurement. BMI is an appropriate measure of nutritional status because it incorporates total body weight and is indexed to the larger population for body weight comparison. Second, longer subacute postoperative nutritional follow-up was not examined.

We conclude that the RGF technique provides a new and likely safer alternative for enteral feeding in patients with esophageal cancer.

## Supporting Information

Video S1 **After the esophagus was reconstructed with a gastric tube inserted retrosternally, a 3.0–5.0-cm linear incision was made in the anterior wall of the stomach under the xiphoid.** A feeding tube (16 Fr. gastric tube; length, 125 cm; diameter, 5.3 mm; TERUMO Medical Products Co., Ltd. Hangzhou, China) was inserted through the incision into the intestinal lumen to a depth of 10 to 25 cm. The feeding tube was secured in place by a double purse-string suture, embedded within the omentum, and the peritoneum was sutured to the gastric wall near the tube. Subsequently, the feeding tube was passed out through the anterior abdominal wall and secured in place.
(AVI)

## References

[1] Myers JG, Page CP, Stewart RM, Schwesinger WH, Sirinek KR, et al (1995) Complications of needle catheter jejunostomy in 2,022 consecutive applications. Am J Surg 170: 547–550 discussion 550-541. 7491998 10.1016/s0002-9610(99)80013-0

[2] Beier-Holgersen R, Boesby S (1996) Influence of postoperative enteral nutrition on postsurgical infections. Gut 39: 833–835. 9038665 10.1136/gut.39.6.833 PMC1383455

[3] Gupta V (2009) Benefits versus risks: a prospective audit. Feeding jejunostomy during esophagectomy. World J Surg 33: 1432–1438. 19387726 10.1007/s00268-009-0019-1

[4] Gerndt SJ, Orringer MB (1994) Tube jejunostomy as an adjunct to esophagectomy. Surgery 115: 164–169. 8310404

[5] Lerut T, Nafteux P, Moons J, Coosemans W, Decker G, et al (2004) Three-field lymphadenectomy for carcinoma of the esophagus and gastroesophageal junction in 174 R0 resections: impact on staging, disease-free survival, and outcome: a plea for adaptation of TNM classification in upper-half esophageal carcinoma. Ann Surg 240: 962–972 discussion 972-964. 15570202 10.1097/01.sla.0000145925.70409.d7 PMC1356512

[6] Altorki N, Kent M, Ferrara C, Port J (2002) Three-field lymph node dissection for squamous cell and adenocarcinoma of the esophagus. Ann Surg 236: 177–183. 12170022 10.1097/00000658-200208000-00005 PMC1422563

[7] Morita M, Yoshida R, Ikeda K, Egashira A, Oki E, et al (2008) Advances in esophageal cancer surgery in Japan: an analysis of 1000 consecutive patients treated at a single institute. Surgery 143: 499–508. 18374047 10.1016/j.surg.2007.12.007

[8] Li H, Yang S, Zhang Y, Xiang J, Chen H (2012) Thoracic recurrent laryngeal lymph node metastases predict cervical node metastases and benefit from three-field dissection in selected patients with thoracic esophageal squamous cell carcinoma. J Surg Oncol 105: 548–552. 22105736 10.1002/jso.22148

[9] Pennathur A, Luketich JD (2008) Resection for esophageal cancer: strategies for optimal management. Ann Thorac Surg 85: S751–756. 18222210 10.1016/j.athoracsur.2007.11.078

[10] Urschel JD, Urschel DM, Miller JD, Bennett WF, Young JE (2001) A meta-analysis of randomized controlled trials of route of reconstruction after esophagectomy for cancer. Am J Surg 182: 470–475. 11754853 10.1016/s0002-9610(01)00763-2

[11] Witzel OI (1891) Sur technik der magenfistelanlegung. Zentralbl Chir 18: 601–604.

[12] Allum WH, Griffin SM, Watson A, Colin-Jones D (2002) Association of Upper Gastrointestinal Surgeons of Great B, (2002) et al Guidelines for the management of oesophageal and gastric cancer. Gut 50 Suppl 5: v1–23. 12049068 10.1136/gut.50.90005.v1 PMC1867706

[13] Bozzetti F, Migliavacca S, Scotti A, Bonalumi MG, Scarpa D, et al (1982) Impact of cancer, type, site, stage and treatment on the nutritional status of patients. Ann Surg 196: 170–179. 7092367 10.1097/00000658-198208000-00009 PMC1352472

[14] Burt ME, Brennan MF (1984) Nutritional support of the patient with esophageal cancer. Semin Oncol 11: 127–135. 6427930

[15] Maxton DG, Menzies IS, Slavin B, Thompson RP (1989) Small-intestinal function during enteral feeding and starvation in man. Clin Sci (Lond) 77: 401–406. 2509127 10.1042/cs0770401

[16] Kudsk KA (2002) Current aspects of mucosal immunology and its influence by nutrition. Am J Surg 183: 390–398. 11975926 10.1016/s0002-9610(02)00821-8

[17] Braga M, Gianotti L, Nespoli L, Radaelli G, Di Carlo V (2002) Nutritional approach in malnourished surgical patients: a prospective randomized study. Arch Surg 137: 174–180. 11822956 10.1001/archsurg.137.2.174

[18] Bozzetti F, Braga M, Gianotti L, Gavazzi C, Mariani L (2001) Postoperative enteral versus parenteral nutrition in malnourished patients with gastrointestinal cancer: a randomised multicentre trial. Lancet 358: 1487–1492. 11705560 10.1016/S0140-6736(01)06578-3

[19] Mochizuki H, Togo S, Tanaka K, Endo I, Shimada H (2000) Early enteral nutrition after hepatectomy to prevent postoperative infection. Hepatogastroenterology 47: 1407–1410. 11100363

[20] Moore EE, Jones TN (1986) Benefits of immediate jejunostomy feeding after major abdominal trauma–a prospective, randomized study. J Trauma 26: 874–881. 3095557 10.1097/00005373-198610000-00003

[21] Aiko S, Yoshizumi Y, Sugiura Y, Matsuyama T, Naito Y, et al (2001) Beneficial effects of immediate enteral nutrition after esophageal cancer surgery. Surg Today 31: 971–978. 11766084 10.1007/s005950170005

[22] Joshi N, Localio AR, Hamory BH (1992) A predictive risk index for nosocomial pneumonia in the intensive care unit. Am J Med 93: 135–142. 1497009 10.1016/0002-9343(92)90042-a

[23] Leibovitz A, Plotnikov G, Habot B, Rosenberg M, Segal R (2003) Pathogenic colonization of oral flora in frail elderly patients fed by nasogastric tube or percutaneous enterogastric tube. J Gerontol A Biol Sci Med Sci 58: 52–55. 12560411 10.1093/gerona/58.1.m52

[24] Cheatham ML, Chapman WC, Key SP, Sawyers JL (1995) A meta-analysis of selective versus routine nasogastric decompression after elective laparotomy. Ann Surg 221: 469–476 discussion 476-468. 7748028 10.1097/00000658-199505000-00004 PMC1234620

[25] Alverdy J, Chi HS, Sheldon GF (1985) The effect of parenteral nutrition on gastrointestinal immunity. The importance of enteral stimulation. Ann Surg 202: 681–684. 3935061 10.1097/00000658-198512000-00003 PMC1250998

[26] Mathus-Vliegen LM, Koning H (1999) Percutaneous endoscopic gastrostomy and gastrojejunostomy: a critical reappraisal of patient selection, tube function and the feasibility of nutritional support during extended follow-up. Gastrointest Endosc 50: 746–754. 10570331 10.1016/s0016-5107(99)70153-7

[27] Swann HM, Sweet DC, Michel K (1997) Complications associated with use of jejunostomy tubes in dogs and cats: 40 cases (1989–1994). J Am Vet Med Assoc 210: 1764–1767. 9187726

[28] Puri V, Hu Y, Guthrie T, Crabtree TD, Kreisel D, et al (2011) Retrograde jejunogastric decompression after esophagectomy is superior to nasogastric drainage. Ann Thorac Surg 92: 499–503. 21704297 10.1016/j.athoracsur.2011.03.082

[29] Nelson R, Edwards S, Tse B (2007) Prophylactic nasogastric decompression after abdominal surgery. Cochrane Database Syst Rev CD004929. 17636780 10.1002/14651858.CD004929.pub3 PMC6669251

